# Comparison of physician networks constructed from thresholded ties versus shared clinical episodes

**DOI:** 10.1007/s41109-018-0084-1

**Published:** 2018-08-13

**Authors:** Jukka-Pekka Onnela, A. James O’Malley, Nancy L. Keating, Bruce E. Landon

**Affiliations:** 1 Harvard T.H. Chan School of Public Health, Harvard University, 655 Huntington Avenue, Boston, MA USA; 2 Department of Biomedical Data Science, The Dartmouth Institute for Health Policy and Clinical Practice, Geisel School of Medicine at Dartmouth, Hanover, NH USA; 3 Department of Health Care Policy, Harvard Medical School, Boston, MA USA; 4 Division of General Internal Medicine, Department of Medicine, Brigham and Women’s Hospital, Boston, MA USA; 5 Division of Primary Care and General Internal Medicine, Department of Medicine, Beth Israel Deaconess Medical Center, Boston, MA USA

**Keywords:** Physician networks, Episodes of care, Network communities, Medicare data

## Abstract

**Objective:**

To compare standard methods for constructing physician networks from patient-physician encounter data with a new method based on clinical episodes of care.

**Data source:**

We used data on 100% of traditional Medicare beneficiaries from 51 nationally representative geographical regions for the years 2005–2010.

**Study design:**

We constructed networks of physicians based on their shared patients. In the fixed-threshold networks and adaptive-threshold networks, we included data on all patient-physician encounters to form the physician-physician ties, and then subsequently thresholded some proportion of the strongest ties. In contrast, in the episode-based approach, only those patient-physician encounters that occurred within shared clinical episodes treating specific conditions contributed towards physician-physician ties.

**Data collection/extraction methods:**

We extracted clinical episodes in the Medicare data and investigated structural properties of the patient-sharing networks of physicians, temporal dynamics of their ties, and temporal stability of network communities across the two approaches.

**Principal findings:**

The episode-based networks accentuated ties between primary care physicians (PCPs) and medical specialists, had ties that were more likely to reappear in the future, and appeared to have more fluid community structure.

**Conclusions:**

Constructing physician networks around shared episodes of care is a clinically sound alternative to previous approaches to network construction that does not require arbitrary decisions about thresholding. The resulting networks capture somewhat different aspects of patient-physician encounters.

**Electronic supplementary material:**

The online version of this article (10.1007/s41109-018-0084-1) contains supplementary material, which is available to authorized users.

## Introduction

The ever-increasing availability of electronic administrative and social interaction data has made it possible to study human behavior at unprecedented scale and depth (Lazer et al. 2009; Onnela et al. 2007). These types of data also provide the opportunity to study the behavior of groups of individuals in different social or organizational contexts, such as a healthcare system, where the outcomes would be expected to be related to the structure of these interactions.

Social networks are a natural way to capture the structure of interactions among a group of people, where a network node corresponds to an individual and a network tie corresponds to a relationship between the two individuals. Administrative data have recently been used to study physician-physician information sharing networks, which consist of individual physicians as nodes with ties defined by patient-sharing relationships among physicians (Landon et al. 2012; Landon et al. 2013; Mandl et al. 2014; Ong et al. 2016; Pollack et al. 2012; Pollack et al. 2013), i.e., networks. At a more fundamental level, however, physician-patient encounter data form a bipartite network consisting of two types of nodes, physicians and patients, and they detail physician-patient interactions from which physician-physician interactions are intuited. The term bipartite refers to any network consisting of two types of nodes such that each edge connects a node from one category with a node from the other category; the bipartite networks studied here form either patient-physician ties or episode-physician ties. These bipartite networks can be projected to unipartite networks consisting of physicians only, where ties reflect patient-sharing between physicians. This standard practice is useful because it enables the study of connections among physicians, but unfortunately the optimal method for identifying meaningful information sharing relationships using administrative data is not clear. For instance, in some cases, such identified relationships between two physicians might be spurious (i.e., not reflective of a true relationships), resulting from a common tie to another physician with whom both physicians share information. For example, a patient may see a primary care physician as well as a gastroenterologist for an ulcer and an ophthalmologist for cataracts. While the primary care physician likely has interactions with both physicians, the gastroenterologist and ophthalmologist are unlikely to interact with each other regarding this patient’s care.

Prior approaches to identifying physician social networks based on shared patients have typically projected all interactions in the underlying bipartite networks to a unipartite network, and have then thresholded ties in the unipartite network based on their strength (i.e., the number of shared patients) to retain the most important connections (Landon et al. 2012). The rationale of connecting physicians via shared patients is the notion that a shared patient is a conduit for the diffusion of clinical practices and information among the physicians who provide care to that patient. While this logic is reasonable if all of the patient-physician encounters are tied to the same episode of care for a particular condition, necessitating at least some information sharing among the physicians about the episode, the patient visits to a host of physicians could be associated with several different clinical episodes, and physicians providing care to a patient in the context of one clinical episode may not need to be informed about the details of another clinical episode. Consequently, if there were little or no transfer of information across different clinical episodes for the given patient, the standard approach would lead one to conclude the network to be denser, or more connected, than is the case. An episode-based network construction, therefore, might offer an improvement over existing methods because it results in less dense networks comprised of physician-physician ties that are expected to have greater likelihood to correspond to real connections among the physicians. The episode-based approach also removes the somewhat arbitrary and unprincipled step of network thresholding. Although statistical arguments can be applied for selecting an optimal threshold, the episode-based network approach avoids the thresholding problem altogether by relying on a different network construction method that focuses on clinically-relevant connections. The end product of the episode-based approach is a network that is a superposition of the connections induced by each individual episode. This approach generalizes one used in some prior research that focused on episodes of care for a particular clinical condition such as prostate cancer or diabetes, that necessarily are limited to physicians treating that condition (Pollack et al. 2014; Pollack et al. 2012; Pollack et al. 2013). Other research has used networks constructed around episodes of care similar to the approach that we take to examine care and outcomes for Medicare beneficiaries undergoing a coronary artery bypass grafting (CABG) (Hollingsworth et al. 2015) (Hollingsworth et al. 2016) (Jordan et al. 2018) (Funk et al. 2017). These papers, however, examined networks constructed around just a single procedure or clinical condition. In contrast, our paper presents a first systematic exploration of the differences between networks constructed from all patient-physician encounters, two different thresholded variants of such networks, and networks constructed around episodes of care. Rather than examining care around a specific episode type, we use data across all episodes and offer these resulting networks as an alternative to constructing networks of all patient-physician encounters with or without thresholding. In addition to examining nodal or dyadic properties, we also examine longitudinal changes in the community structure of these networks, which can be used to identify and study the evolution of teams of physicians that provide care to their patients.

In this paper, we use administrative data from the Medicare program to construct physician networks for 51 hospital referral regions (HRRs) in the US using these two different approaches, the standard patient-based network construction method (Landon et al. 2012) and a new episode-based network construction method introduced here. We characterize the differences in the networks in terms of some commonly used network summary statistics. Instead of examining only a single network snapshot (or cross-section), we construct a sequence of networks, based on annual data from 2005 to 2010. In addition to descriptive analyses at the level of network ties, we identify longitudinal network communities and characterize the stability of community structure over time in these annual “multi-slice” networks obtained using the two different methods of network construction. Network communities can be defined as sets of nodes that are densely connected to other nodes in the same community but only sparsely to nodes in other communities. In our setting, network communities are potentially relevant units of analysis because they go beyond individual physician pairs and identify “clusters” of connected node pairs within the networks, i.e., groups of physicians that jointly provide care to patients.

## Methods

### Data source

We used data on 100% of traditional Medicare beneficiaries from 51 hospital referral regions (HRRs) for the years 2005–2010. A total of 50 HRRs were randomly sampled with probability proportional to their size; the 51st HRR was Boston, which was included because of our familiarity with it. This was the maximum amount of data that we were permitted to purchase. We defined encounters with physicians based on paid claims in the carrier file. We excluded claims for non-direct patient care specialties or specialties where individual physicians are not typically selected by patients (e.g., anesthesia, radiology). We identified all evaluation and management services, and included procedures with a relative value unit (RVU) value of at least 2.0 in order to capture surgical procedures that often are reimbursed via bundled fees that include pre- and post-procedure assessments. We excluded claims for laboratory and other services not requiring a physician visit; we also excluded claims generated from physicians who saw fewer than 30 Medicare patients during any year or who practice outside of the included HRRs.

### Identifying episodes of care

We identified discreet episodes of care using Symmetry Episode Treatment Groups (ETG), version 8.3 (Optum, Eden Prairie, Minnesota), which is in widespread use nationally. Each episode of care groups clinically related services delivered to a patient with a specific condition over a defined period of time into one of about 600 different episode types, which reflect treatment for both chronic diseases (e.g., diabetes) and acute conditions (e.g., pneumonia, ankle fracture). A total of 92.2% of patient visits are assigned to episodes, and 46.5% of episodes have more than 1 visit associated with them. Each episode has a start date, end date, and a unique episode number. The start date is defined as the first visit date of the episode and usually is preceded by a “clean” period of varying length during which no related encounters occurred. The end date is defined as the last visit date of the episode. Acute episodes are defined as episodes that are expected to resolve and the end date is defined after no additional related visits have occurred for a defined period of time, which varies by type of episode. Note that the start and end dates of acute episodes are distinct from the time windows used to construct the networks. This is in contrast to chronic episodes, which are defined to last an entire calendar year for each year when present.

### Network construction

**Fig. 1:**
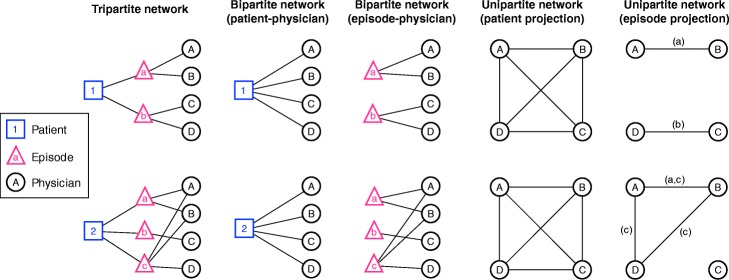
Schematic of different network types associated with physician encounter data. Here the underlying physician visit sequence for Patient 1 is A, B, C, D, and these visits are associated with episodes a, a, b, b, respectively; the physician visit sequence for Patient 2 is A, B, C, A, C, D, B, and the associated episode sequence is a, a, b, c, b, c, c, respectively. The tripartite network connects a patient to one or more episodes, and each episode in turn is connected to one or more physicians. Tripartite networks provide the most complete presentation of the data and preserve all relevant information for network construction. The tripartite network can be projected to three different bipartite networks, each generated by omitting one node type from the tripartite network; here two bipartite networks are shown, one connecting patients and physicians and the other connecting episodes and physicians. Finally, any bipartite network can be projected to two different types of unipartite networks containing nodes of only one type. Here we show one projection for each bipartite network, the projections where the remaining nodes are physicians who are connected either by shared patients or shared episodes. Because of the organization of the tripartite network, we stress that all physician-physician ties are induced by shared patients, but the episode-based approach stipulates the additional constraint that only patients shared within episodes should count

All networks are constructed using 1-year time windows. To construct **episode-based bipartite networks**, for each HRR, we consider a sequence of *physician-episode* pairs that occurred during the time window, where each episode consists of one patient and one or more physicians who provided care to the patient during the given medical episode. To construct **patient-based bipartite networks**, for each HRR, we consider a sequence of *physician-patient* encounters that took place during the entire calendar year. Importantly, there is no requirement that the physicians provide care to a patient for a particular clinical condition (episode), and therefore the latter approach results in a set of physician-physician ties that is a superset of the ties induced by the former approach. For both methods, after constructing bipartite graphs, for each HRR and each calendar year, we construct corresponding **unipartite networks of physicians** by projecting the underlying bipartite networks consisting either of physician-episode ties or physician-patient ties. Intuitively, patient *i* (or episode *i*) in a bipartite network having degree *k*_*i*_ induces a clique of *k*_*i*_ nodes among the physicians he or she is connected to, where a *k*-clique is a fully connected subgraph of *k* nodes and *k*(*k* − 1)/2 edges; the projection of the entire bipartite graph is then the summation of the cliques induced by all patients (or all episodes), where each patient (each episode) contributes precisely one clique to the projected unipartite graph. See Fig. 1 for a schematic on network construction.

The resulting two unipartite graphs for any given HRR and year typically have different numbers of edges, making it difficult to interpret differences in network properties between them. In particular, the patient-based network graphs have many more edges than the episode-based network graphs, and it is likely that those with the lowest numbers of shared patients do not represent true information sharing ties(Barnett et al. 2011). We therefore threshold the patient-based unipartite networks in one of two ways. First, we maintained ties in the top 80% of ties (as defined at the individual node) based on their strength (quantified as the number of shared patients), an approach that we have used previously (Landon et al. 2012; Landon et al. 2013). Second, we created an adaptive threshold for each HRR by removing ties from weakest to strongest until the number of ties remaining matched to within 1% of the number of ties in the corresponding episode-based network. We call the former **fixed-threshold networks** and the latter **adaptive-threshold networks**. Note that both networks are unipartite since they consist of physicians only.

### Tie types

Using physician specialty data from the American Medical Association physician Masterfile, we characterized each physician, and therefore each node, as a primary care provider (P), medical specialist (M), or surgical specialist (S). Since each tie, by definition, consists of a pair of physician nodes, we can stratify all ties into six categories: P-P, S-S, M-M, P-S, P-M, and S-M. For example, a tie between a medical specialist and a primary care physician would be placed in the P-M category (directionality does not matter, this category simply represents a pairing of physicians of certain designations). We use *L*_*XY*_ to denote the number of ties between a provider of type *X* and a provider of type *Y* in a given HRR and a given time window; for example, *L*_*PM*_ stands for the number of ties in a given network between primary care physicians and medical specialists. We use *L* to denote the total number of ties in the given network, and define the proportions of tie types as *l*_*XY*_ = *L*_*XY*_/*L*; for example, here *l*_*PM*_ stands for the proportion of network ties that connect primary care physicians and medical specialists in a network for a given HRR and given time window.

### Tie persistence and reappearance

Tie persistence refers to the lifetime of a tie in a sequence of networks constructed over consecutive time windows (annual in this case) for a given HRR. For simplicity, consider two graphs *G*(*t*_1_) and *G*(*t*_2_) constructed for two consecutive years, say for years 2008 and 2009, respectively. We compute **tie persistence** by first considering the number of ties that exist in both graphs (a tie exists in both if it connects the same two physicians in each graph) in each of the six categories of specialty combinations, and we then divide these numbers by the number of ties in each category in the first graph in the sequence, here *G*(*t*_1_). In a longer sequence of graphs, for a tie to persist it must be present in all graphs in the sequence. A concept related to tie persistence is that of **tie reappearance**. Given a longer sequence of annual graphs, some ties may persist for a few years, then disappear, only to reappear in a later year. Because a tie can only reappear after it has disappeared, the number of tie reappearances is either equal to, or one less, than the number of tie disappearances. Because the observations (ties) may reappear later, censoring of observations is not an issue in this analysis.

### Network communities

To learn about structural properties of the episode-based and patient-based networks at the mesoscopic scale, the scale between microscopic (e.g., node degree) and macroscopic (e.g., graph diameter) structural properties, we use community detection (Fortunato 2010; Newman 2012; Porter, Onnela, and Mucha 2009) to identify groups of densely connected nodes in the networks. In our setting, network communities can be seen to correspond to groups of physicians who jointly provide care to a group of patients. We use a variant of a popular method of modularity maximization (Newman 2006), the so-called multi-slice method (Mucha et al. 2010), which makes it possible to detect network communities in a sequence of temporally ordered networks. This means that rather than detecting network communities separately and independently in each network corresponding to a given HRR and time window, the network communities can now extend over various network slices (time points—years in our case) for each HRR. For example, it is possible for two separate communities to merge together at some point and, similarly, an existing community may split into two or more communities. Community detection is a difficult mathematical and computational problem that requires special software. We refer the reader to the supplement for more details. To quantify the stability of communities over time in each HRR, we used a metric that is related to the entropy of a node’s community assignment in each year (see Additional file 1). For example, if a node is assigned to the same community in each of the six annual slices (in the sequence of six networks corresponding to the six years of data under consideration), it is said to have minimal normalized entropy regarding its community assignment, whereas if a node is assigned to a different community each year, it has maximal normalized entropy in this regard. In simplified terms, entropy can be considered to measure the variability of a node’s community assignment from year to year. Communities that are stable in time may correspond to groups of physicians who provide coordinated care to patients over longer periods of time without significant churn in their membership, i.e., both the group of physicians and the structure of relationships among them remain stable over time.

## Results

### Tie types

Table 1 shows descriptive statistics for the episode-based and the three versions of the patient-based networks from 2005 to 2010 constructed from annual time windows. The episode-based networks (“Episode”, 1st block in the table) include all relevant ties from shared episodes, with no threshold. The full patient-based network (“Patient”, 4th block in the table) includes all patient-sharing ties and has also not been thresholded. The two other types of patient-based networks incorporate thresholds: the adaptive-threshold patient networks (“Patient (A)”, 2nd block in the table), where ties have been removed from weakest to strongest until the number of remaining ties matched to within 1% of the number of ties in the corresponding (same year, same HRR) episode-based network, and the fixed-threshold patient networks whose weights have been thresholded at the 80th percentile (“Patient (80%)”, 3rd block in the table) for each node, the approach we have followed previously. For each network, the number of nodes and ties increases over time, the latter more than the former, leading to increasing average degree over time. Average degrees are essentially identical across episode-based and adaptive-threshold networks by construction (small discrepancies are due to non-uniqueness of edge weights in the thresholding process), but these average degrees are about 30% higher than for networks thresholded at the 80th percentile. Clustering coefficients however are very similar across the first three network types; as expected, non-thresholded networks have substantially higher degree and much higher average clustering coefficient. The proportion of different tie types is essentially identical across adaptive-threshold networks and fixed-threshold networks (fixed 80th percentile threshold), so our subsequent analyses focus on differences between episode-based networks and adaptive- threshold patient networks (the first two blocks in the table). As is clear from the table, episode-based networks contain a greater proportion of ties among medical specialist (roughly 38% vs. 30%) and fewer PCP – medical specialist ties (roughly 12% vs. 19%). In general, episode networks appear to accentuate ties among PCPs and ties among medical specialists, giving less weight to other tie types.

**Table 1:**
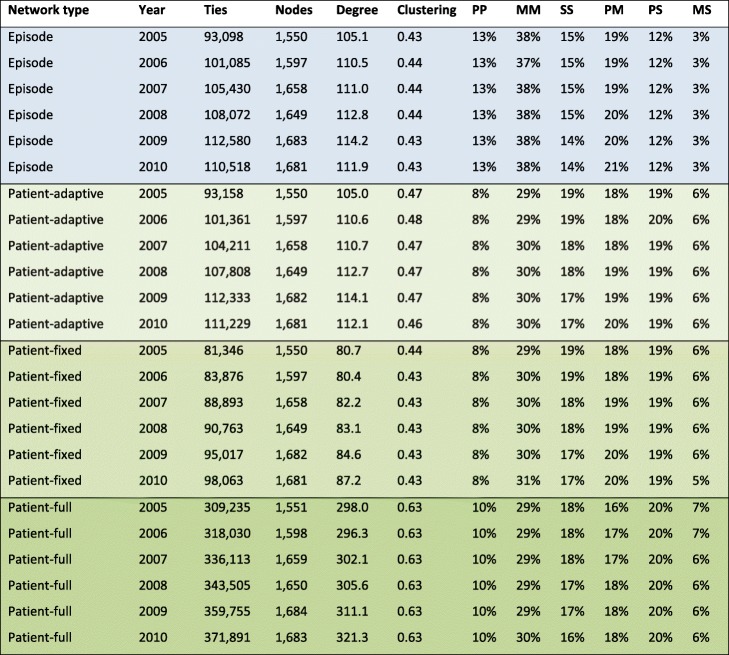
Descriptive statistics for episode-based and patient-based networks from 2005 to 2010 constructed from 1-year windows. All statistics are averages over all HRRs. We report the average number of ties (Ties), average number of nodes (Nodes), average degree (Degree), average clustering (Clustering) and average proportion of tie types based on specialty (last six columns: PP, MM, SS, PM, PS, MS), where the specialties are primary care (P), medical specialist (M), and surgical specialist (S)

### Tie persistence and reappearance

**Fig. 2:**
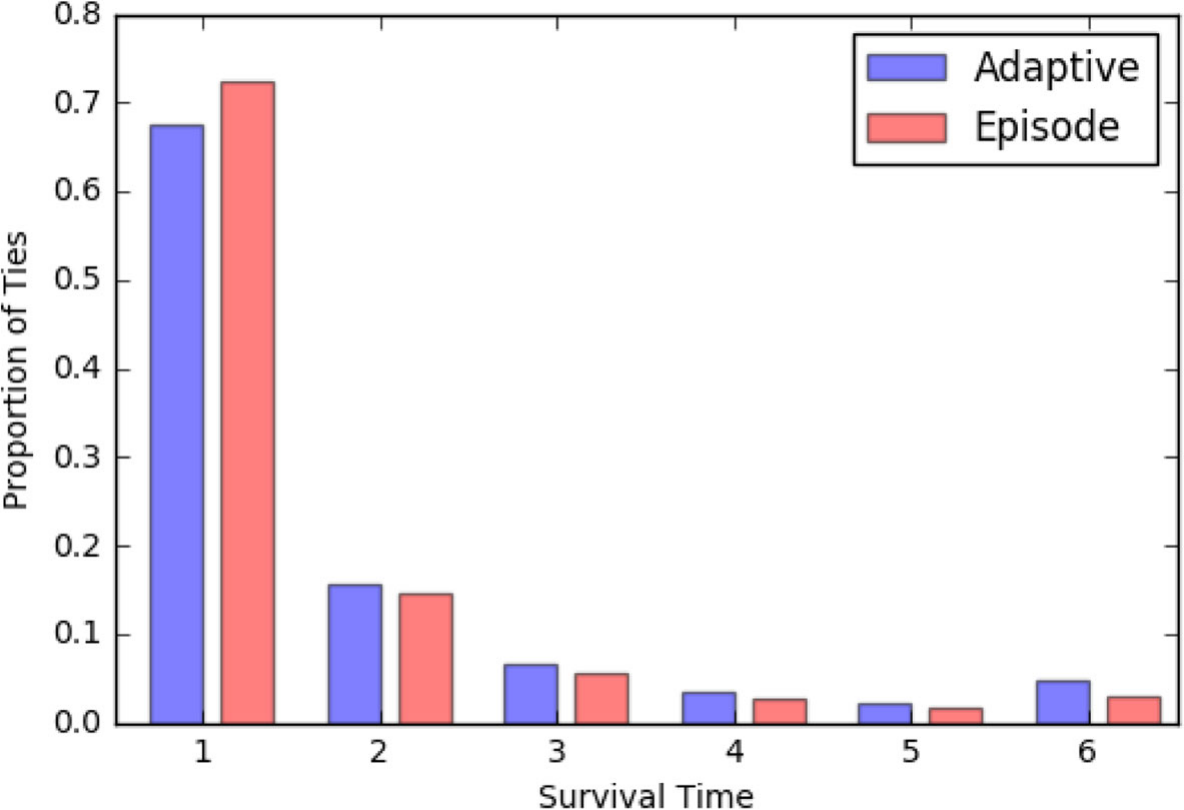
Distribution of tie survival times for patient-based adaptive networks and episode-based networks (no thresholding), the two of the three approaches studied that result in networks with the same number of edges, making them directly comparable to one another. Proportion of surviving ties would be expected to decrease for survival times 1–5. Ties that have survival times equal to 6 survive throughout the studied period

Figure 2 shows the distribution of tie survival times in adaptive-threshold networks and episode-based networks. The distributions are similar overall, although the proportion of ties that persist for only 1-year is greater in episode-based networks (72.4% vs. 67.4%). These numbers are comparable to a recent study using Medicare data and the Dartmouth Atlas, which found that that 70.7% of ties between PCPs and other physicians that were present in 2012 persisted in 2013, and additional shared patients in 2012 increased the odds of being connected in 2013 (DuGoff et al. 2017). Thus, tie persistence time in episode-based networks, 1.56 years, is somewhat smaller than mean tie persistence time in adaptive-threshold networks (1.72 years). In contrast, taken over all HRRs and all years, there were a total of 2,744,224 tie reappearances events in the episode-based networks and 2,437,351 in the adaptive networks. Since our data cover 6 years and 51 HRRs, to make these numbers more interpretable, we compute the average number of tie reappearances per HRR per year which is 8968 in the former category and 7965 in the latter. This demonstrates that there were 12.6% more tie reappearance events in the episode-based networks, suggesting that in those networks ties are more likely to reappear around specific episodes of patient care, thus leading to greater overall persistence over time.

### Stability of network communities over time

**Fig. 3:**
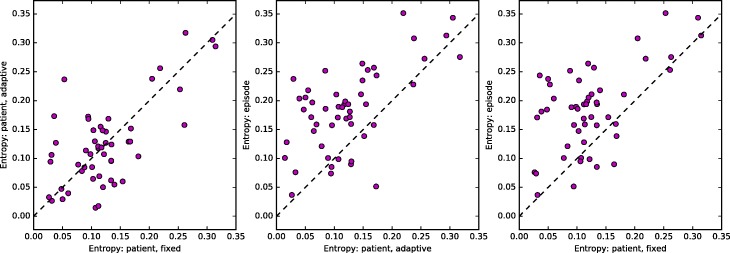
Mean entropy for each HRR, computed over six 1-year windows, using each of the three different ways to construct the network: fixed-threshold, adaptive-threshold, and episode-based. Each panel shows a scatter plot of two of the three entropy measures plotted against one another: fixed-threshold vs. adaptive-threshold (left), adaptive-threshold vs. episode-based (middle), and fixed-patient vs. episode-based (right). The Pearson correlation coefficients and their p-values for testing the hypothesis of no correlation are 0.66 (*p*=1.159e-07), 0.61 (*p*=1.804e-06), and 0.58 (*p*=6.925e-06), respectively

Figure 3 shows the three pairwise plots of normalized entropies computed for fixed-threshold, adaptive-threshold, and episode-based networks. Each dot represents an HRR, and in general the mean entropies are reasonably strongly correlated. The plots also indicate that while normalized entropies for fixed-threshold and adaptive-threshold networks are comparable, episode-based networks have somewhat higher entropy values. We summarize the distribution of normalized entropy for the three ways of constructing networks in Table 2. Median entropy for fixed-threshold and adaptive-threshold networks is comparable at 0.114 and 0.118, respectively, whereas the corresponding value for episode-based networks is 0.189. Since adaptive-threshold networks and episode-based networks have the same number of edges for each HRR, their values are not confounded with the network density, and we still find that episode-based networks have 60% higher normalized entropy than adaptive-threshold networks.

**Table 2:** Summary statistics for the distribution across HRRs of mean entropy (taken over six 1-year windows) for the three different ways of constructing networks

Entropy/Summary	Min	Max	Mean	SD	Median
Fixed-threshold	0.027	0.315	0.124	0.068	0.114
Adaptive-threshold	0.015	0.317	0.124	0.072	0.118
Episode	0.037	0.352	0.185	0.072	0.189

One potential confounder in this latter analysis is that the number of communities detected varies for each network type. The median number of communities for fixed-threshold networks is 10 (range 4–20), for adaptive-threshold networks 9 (range 3–20), and for episode-based networks 14 (range 7–21). The larger number of communities in episode-based networks could potentially inflate the observed entropy values because there are more communities in which to be assigned. To investigate this, we regressed normalized entropy against the number of communities for each of the three network types using linear models, resulting in three separate models. We estimated the value of entropy by setting the predictor, the number of communities, to the mean number of communities observed in the adaptive-threshold networks (9.7), which resulted in entropy values of 0.12 (95% CI: 0.10–0.14) for fixed-threshold networks, 0.12 (CI: 0.10–0.14) for adaptive-threshold networks, and 0.17 (CI: 0.14–0.20) for episode-based networks. We also estimated these numbers at the mean number of communities observed in the episode-based networks (13.7), which resulted in entropy values of 0.15 (95% CI: 0.13–0.18) for fixed-threshold networks, 0.15 (CI: 0.11–0.18) for adaptive-threshold networks, and 0.19 (CI: 0.17–0.21) for episode-based networks. Therefore, the episode-based approach results in greater values for entropy whether we compute the expected entropy at the mean number of communities in the adaptive-threshold networks or at the mean number of communities in the episode-based networks. This suggests that increased entropy is a characteristic of networks constructed using only patient-physician encounters within shared episodes of care.

## Discussion

We compared three different methods of constructing patient-sharing networks of physicians, which we termed fixed-threshold networks, adaptive-threshold networks, and episode-based networks. Comparing adaptive-threshold networks and episode-based networks, which had the same number of ties, we found that episode-based networks retained more PCP-PCP ties and specialist-specialist ties. Ties in both types of networks appeared and disappeared in time, but in episode-based networks ties had somewhat lower persistence times and ties that had existed in the past were more likely to reappear in the future.

Weak ties present a special challenge to all approaches to network construction, a problem that is aggravated by the presence of what is effectively measurement noise. It is a common finding that large-scale weighted networks have strongly right-skewed tie strength distributions, meaning that most network ties are weak. Applying a single relatively large threshold to patient-based ties will eliminate most if not all of them, and many of these will be true information sharing relationships. In contrast, the episode-based approach applied without thresholding, as we have done here, is guaranteed to preserve all ties, including weak ties, that pertain to the episodes of interest. The tradeoff between (thresholded) patient-based and (non-thresholded) episode-based networks, as regards weak ties, is that the former will have many false negatives but few false positives, whereas the latter may have some false positives but few false negatives. Our observations regarding lower persistence but higher reappearance of ties suggest that episode-based networks may be more sensitive to detecting ties that are weak but meaningful. Because episode-based ties are not thresholded, they may be based on few or even just a single shared patient and would thus be expected to decay faster over time. However, to the extent that these weak ties reflect genuinely important interactions among physicians, we would expect those ties to reappear should to opportunity arise.

We also studied longitudinal network communities, examining their stability using an entropy-based metric. We focused on network communities because they arguably correspond to functional groupings of physicians and therefore could directly affect delivery of care. Although the changes in network structure at the level of individual ties were not very large, their net effect was that communities in episode-based networks were more dynamic. Measured by their relative entropy, episode-based networks had 60% greater normalized entropy than adaptive-threshold networks. This finding appears to be at least in part due to episode-based networks having more communities, but even after a simple adjustment for the number of communities, episode-based networks had greater entropy. Part of the explanation almost certainly has to do with the persistence and reappearance of ties, but it is possible that there are additional factors, such as the extent to which tie appearance and disappearance events are correlated in different regions of the network, that give rise to this outcome.

The fundamental reason for some type of network thresholding arises from the fact that non-thresholded networks tend to be very densely connected and also include ties that are likely to be spurious. This a problem from the point of view of interpretation, as it is unlikely that weak or sporadic ties are significant conduits of information, but it additionally presents challenges for computing some of the commonly used network metrics and measures. Our past work has made use of the fixed-threshold approach to arrive at sparser networks, but his approach requires imposing a relatively arbitrary threshold, which might not maximize the number of true information sharing ties maintained. An important benefit to the episode-based approach is that it provides a solution to the thresholding problem that is based on clinical interactions and theory rather than a relatively arbitrary cut point. Across all HRRs, the episode-based networks (and therefore the adaptive-threshold networks) eliminated on average 70.6% of all edges, effectively retaining the top 30% of the strongest and most clinically relevant physician-physician ties.

Although it is possible to consider the thresholding problem from statistical grounds, where it could be cast as a variable selection problem with the ties corresponding to variables, episode-based networks offer a domain-specific approach to the thresholding problem. Moreover, episode-based networks are clinically intuitive and the retained ties are more likely to correspond to information-sharing ties. Notwithstanding their face validity, episode-based networks appear to have more dynamic community structure, which presents some challenges for interpretation. One possible reason for this is that thresholded networks retain connections among high-volume providers, leading to fewer communities and falsely stabilizing network structure over time.

Our study has strengths and limitations. A key strength of our paper is the use of Medicare patient-physician encounter data and data on episodes of care to compare different ways of constructing physician-physician networks. Another strength is the examination of the resulting networks not only at the level of individual physicians, but also at the level of groups of physicians, here conceptualized as network communities, as well as the extension of this analysis to multiple years of data. Because we detected the network communities using a specific method, it is possible that other approaches could yield different results, although lack of longitudinal community detection methods prevents a more thorough investigation of this possibility. Our analyses were also confined to the 51 HRRs for which data were available and included only fee-for-service Medicare patients, but given that 50 of the included HRRs were randomly sampled from all HRRs (the 51st HRR, Boston, was included by design because of our familiarity with it), we expect our results to generalize to all HRRs. For the included HRRs, we had a 100% sample of traditional Medicare beneficiaries. The software that we used to generate the episodes of care is proprietary, which potentially makes replication of these results more difficult, but this problem can be alleviated by using a public domain episode grouper (Centers for Medicare & Medicaid Services 2011).

Medicare patient-physician encounter data continue to present vast opportunities for investigating provision of medical care. The size and complexity of these data present many challenges for data analysis and substantive interpretation. The episode-based approach we have introduced in this paper is informed by clinical intuition and uses a theory-driven approach wherein we require the two physicians to be linked through shared care of an episode for a particular patient. This allows us to eliminate ties that likely are spurious without requiring an arbitrary threshold to be assigned. These factors make the episode-based method preferable to other approaches in most research settings. In addition, the episode-based approach allows for a much more generalized approach to network construction; for example, as some prior studies have done, one could base these networks on specific types of episodes, which might be helpful, for instance, for studying specific procedures or conditions, such as cancer (Pollack et al. 2014; Pollack et al. 2012; Pollack et al. 2013). These types of analyses are not a potential extension of the standard thresholding approach but rather a restriction that could be applied on conjunction with an episode-based approach. Fundamentally, we see the episode-based approach, compared to the patient-based approach, as giving rise to measurements that are less contaminated with noise, and therefore these more precise measurements of physician-physician interactions are more likely to reveal the true underlying structure of these networks. In conclusion, filtering networks by clinical episodes of care is a step towards making research that uses these data more powerful, understandable and actionable for patients and physicians.

## Additional file

Additional file 1: Supplementary material. (DOCX 20 kb)

## Abbreviations

ETG: Episode Treatment Groups
HRRs: Hospital Referral Regions
PCP: Primary Care Physician
RVU: Relative Value Unit
