# Supplementary material for: Comparison of physician networks constructed from thresholded ties versus shared clinical episodes
Source: Appl Netw Sci. 2018 Aug 13;3(1):28. doi: 10.1007/s41109-018-0084-1 (PMC6214299; doi:10.1007/s41109-018-0084-1)
Supplement: Supplementary file 1 — Supplementary material. (DOCX 20 kb) [file 41109_2018_84_MOESM1_ESM.docx]

**Supplementary Material**

Table 1. Mean percentages of ties thresholded in adaptive-threshold networks to match the number of ties in the corresponding episode-based networks for each HRR. The thresholding is done separately for each 1-year window in each HRR, and the reported numbers are means computed across all years. The mean of all entries in the table, i.e., mean across all HRRs across all years, is 70.6%.

| 5 | 7 | 12 | 25 | 79 | 82 | 101 | 111 | 123 |
| --- | --- | --- | --- | --- | --- | --- | --- | --- |
| 70.8 | 69.5 | 76.6 | 67.4 | 73.2 | 75.8 | 71.9 | 75.0 | 72.7 |
| 127 | 133 | 137 | 140 | 156 | 164 | 175 | 183 | 185 |
| 78.6 | 63.5 | 72.9 | 68.5 | 76.3 | 62.0 | 66.1 | 76.3 | 62.8 |
| 196 | 204 | 208 | 209 | 210 | 213 | 216 | 227 | 232 |
| 62.2 | 74.2 | 64.8 | 63.5 | 72.9 | 69.5 | 76.0 | 78.6 | 76.3 |
| 250 | 253 | 256 | 274 | 277 | 281 | 293 | 299 | 303 |
| 67.7 | 78.1 | 69.0 | 69.0 | 63.8 | 74.7 | 74.5 | 77.1 | 81.5 |
| 309 | 324 | 341 | 342 | 351 | 352 | 359 | 374 | 376 |
| 71.1 | 64.1 | 66.7 | 69.0 | 67.7 | 72.7 | 61.7 | 62.8 | 67.7 |
| 379 | 400 | 423 | 428 | 430 | 443 |  |  |  |
| 75.3 | 71.4 | 78.6 | 58.9 | 72.1 | 69.3 |  |  |  |

**Methods**

**Episode-based graphs.** To construct episode-based bipartite graphs, for each HRR, we consider a sequence of physician-episode pairs that took place during the given time window, where each episode consists of one patient and one or more physicians who provided care to the patient during the given medical episode. For each HRR, in the given time window, we construct a bipartite graph with episode-based incidence matrix $B^{E}$ such that $B_{ij}^{E}$ corresponds to the number of times physician $i$ provided care to a patient during episode $j$.

**Patient-based graphs.** To construct patient-based bipartite graphs, for each HRR, we consider a sequence of physician-patient encounters that took place during the given time window. For each HRR, in the given time window, we construct a bipartite graph with patient-based incidence matrix $B^{P}$ such that $B_{ij}^{P}$ corresponds to the number of times physician $i$ provided care to patient $j$.

**Network projection.** For each HRR, we construct unipartite graphs of physicians by projecting the underlying bipartite graphs consisting either of physicians and episodes in episode-based networks or of physicians and patients in patient-based networks. Using standard terminology and notation, we use $A$ to denote the adjacency matrix of a unipartite graph consisting of physicians. Given that we use two methods to construct the networks, we have two bipartite incidence matrices $B^{P}$ and $B^{E}$, and we therefore obtain two unipartite adjacency matrices ${A^{P}=B}^{P}{{(B}^{P})}^{T}$and ${A^{E}=B}^{E}{{(B}^{E})}^{T}$, where the superscript T indicates matrix transpose, and where we set the diagonal elements of the resulting adjacency matrices to zero. Intuitively, patient $i$ (or episode $i$) in a bipartite network having degree $k_{i}$ induces a clique of order $k_{i}$ among the physicians he or she is connected to, where a clique of order $k$ consists of $k$ nodes and $\binom{k}{2}=k(k-1)/2$ edges; the projection of the entire bipartite graph is then the superposition of the cliques induced by all patient (or all episodes), where each patient (each episode) contributes precisely one clique. The resulting projected unipartite graphs, for a given HRR, have typically different number of edges, making it hard to compare many network properties. We therefore threshold the patient-based unipartite graph, using the bisection method, until the percentage of ties present in it matches (to within 1%) the percentage of ties present in the corresponding episode-based network.

**Episodes and time windows.** It is possible for acute episodes to cross the boundary between two consecutive windows, and since our windows are defined as calendar years, these episodes can cross the boundary between calendar years. Consider first the window-crossing scenario for patient-based (rather than episode-based) networks. Here we derive a temporally ordered visit sequence of physicians for each patient, and we form a clique among those physicians (in the projected unipartite network of physicians) who provided care to the patient during visits that have visit dates within the time window of interest (start date inclusive, end date exclusive). The width of the aggregation time window will impact the structure of the networks for patient-based networks, but nevertheless accurately reflects the structure of care within those time windows. Considering next the window crossing scenario for episode-based networks, here we again derive a temporally ordered visit sequence of physicians, this time not for each patient but for each episode. (Note that because each episode is assigned a unique identifier, it is natural to think of episodes as clustered within a patient.) We now form a clique among those physicians who provided care to the patient during visits that occurred within that episode and that have visit dates within the time window of interest. It is possible, in fact expected, that some visits pertaining to a given episode will fall to the end of one year and the beginning of next. For example, assume that a patient saw five physicians within an episode, and label these physicians, in the order in which they were seen, A, B, C, D, and E. If the first two visits within the episode fall within one year and the next three visits within the next, then the patient will have encountered physicians A and B in the former year and physicians C, D and E in the latter. In this scenario, we would connect physicians A and B in the former year (physicians C, D, and E not being present) and we would connect physicians C, D and E in the latter year (physicians A and B not being present. These networks will accurately reflect the structure of care within episodes within those time windows. Because acute episodes have limited duration, the resulting networks are not particularly sensitive to the choice of window width.

**Tie persistence and reappearance.** Tie persistence refers to the lifetime of a tie in a sequence of networks constructed from consecutive time windows. Let $T$ denote the width of a time window used to construct any given graph and let $\delta T$ denote the temporal displacement between any two consecutive windows. Consider two graphs $G(t_{1})$ and $G(t_{2})$ and define corresponding sets of specific tie types in these graphs as $S_{XY}(t_{1})$ and $S_{XY}(t_{2})$. We define persistence of ties of a given type as $|\bigcap_{t=t_{1}}^{t=t_{2}} S_{XY}\left( t \right)|/|S_{XY}(t_{1})|$, in other words, we first construct the set of ties that exist in every graph in the sequence from $G(t_{1})$ to $G(t_{2})$ by using set intersection and subsequently divide the number of edges in that set by the number of ties of that type in the graph $G(t_{1})$. Some ties may persist for a few time steps, disappear, and then reappear later in the network. We refer to this process as tie reappearance, and because a tie can only reappear after it has disappeared, the number of tie reappearances is either equal to, or one less, than the number of tie disappearances. (Note that the one less case corresponds to a tie not reappearing again in a sequence, or a tie persisting to the end of data, leading to right censoring.)

**Detecting network communities.** To learn about structural properties of the episode-based and patient-based networks at the mesoscopic scale, the scale between microscopic (e.g., node degree) and macroscopic (e.g., graph diameter) structural properties, we use community detection to identify groups of densely connected nodes in the networks. More specifically, we use a variant of a popular method of modularity maximization, the so-called multi-slice method, which makes it possible to detect network communities in a sequence of temporally ordered networks. The community assignments are determined by maximizing the following quality function over different assignments of node-slices (a given node within a given slice) to communities:

$Q_{MS}=\frac{1}{2\mu}\sum_{i,j,s,r} \left[ \left( A_{ijs}-\gamma_{s}\frac{k_{is}k_{js}}{2m_{s}} \right)\delta_{sr}+\delta_{ij}C_{jsr} \right]\delta(g_{is},g_{jr})$.

Here $A_{ijs}$ is the adjacency between node $i$ and node $j$ in slice $s$, $C_{jsr}$ is the strength of coupling for node $j$ between slices $s$ and $r$, $\gamma_{s}$ is the so-called resolution parameter for slice $s$, $\delta$ is the Kronecker delta, $k_{is}$ is the degree of node $i$ within slice $s$, $\mu$ is a normalization term. All of these quantities are fixed either by network structure or by the choice of tuning parameters, which here are $\gamma_{s}$ and $C_{jsr}$, where the latter is set to $\omega$ for all nodes if slices $s$ and $r$ and contiguous in time, otherwise it is set to 0. The maximization of $Q_{MS}$ takes place by modifying the community assignments of nodes, which here are denoted with $g_{is}$ for node $i$ in slice $s$. In practice, $Q_{MS}$ is maximized by using suitable heuristics, and here we settle for the so-called Louvain method. To reiterate, a community partition consists of finding suitable values for the variables $g_{is}$ for each node $i$ and for each slice $s$; community assignments can be interpreted as categorical variables such that $g_{is}=1,\ldots,m$ where $m$ is the number of communities in the multi-slice system for the given HRR. Note that the value of $m$, the number of communities, is not pre-specified but, instead, the methods determines that automatically.

**Stability of network communities over time.** We detect communities using this approach in each HRR using 1-year windows for network construction, which resulted in six slices in each HRR, and we repeat this process for unipartite networks projected from both episode-based and patient-based networks. To quantify the stability of communities, we first compute entropy of community assignments for each node, so that for a given HRR, the entropy of node $i$ is given by $s_{i}=-\sum_{k} p_{k}\ln p_{k}$ where $p_{k}$ is the probability that a node is assigned to community $k$. For example, if a node is assigned to the same community in each of the six slices, such that the vector $g_{i}=\left[ g_{i1},\ldots, g_{i6} \right]^{T}=\left[ 2,2,2,2,2,2 \right]^{T}$, this assignment leads to minimal entropy, whereas say if $g_{i}=\left[ 5,3,1,4,6,8 \right]^{T}$, the assignment leads to maximal entropy. Instead of using entropy directly, we computed normalized entropy $\tilde{s}_{i}=(s_{i}-s_{min})/(s_{max}-s_{min})$, where $s_{min}$ and $s_{max}$ are the minimum and maximum entropies possible for the given number of slices. All results for entropy reported in the main paper are in terms of normalized entropy $\tilde{s}_{i}$.
